# Mental health and well-being during the second wave of COVID-19: longitudinal analyses of the UK COVID-19 Mental Health and Wellbeing study (UK COVID-MH)

**DOI:** 10.1192/bjo.2022.58

**Published:** 2022-06-01

**Authors:** Karen Wetherall, Seonaid Cleare, Heather McClelland, Ambrose J. Melson, Claire L. Niedzwiedz, Ronan E. O'Carroll, Daryl B. O'Connor, Steve Platt, Elizabeth Scowcroft, Billy Watson, Tiago Zortea, Eamonn Ferguson, Kathryn A. Robb, Rory C. O'Connor

**Affiliations:** Suicidal Behaviour Research Laboratory, Institute of Health & Wellbeing, University of Glasgow, UK; Suicidal Behaviour Research Laboratory, Institute of Health & Wellbeing, University of Glasgow, UK; Suicidal Behaviour Research Laboratory, Institute of Health & Wellbeing, University of Glasgow, UK; Suicidal Behaviour Research Laboratory, Institute of Health & Wellbeing, University of Glasgow, UK; Institute of Health & Wellbeing, University of Glasgow, UK; Division of Psychology, University of Stirling, UK; School of Psychology, University of Leeds, UK; Usher Institute, University of Edinburgh, UK; Samaritans, UK; Scottish Association for Mental Health, UK; Oxford Institute of Clinical Psychology Training and Research, University of Oxford, UK; School of Psychology, Nottingham University, UK; Institute of Health & Wellbeing, University of Glasgow, UK; Suicidal Behaviour Research Laboratory, Institute of Health & Wellbeing, University of Glasgow, UK

**Keywords:** COVID-19, mental health, general population, depression, suicidal ideation

## Abstract

**Background:**

Waves 1 to 3 (March 2020 to May 2020) of the UK COVID-19 Mental Health and Wellbeing study suggested an improvement in some indicators of mental health across the first 6 weeks of the UK lockdown; however, suicidal ideation increased.

**Aims:**

To report the prevalence of mental health and well-being of adults in the UK from March/April 2020 to February 2021.

**Method:**

Quota sampling was employed at wave 1 (March/April 2020), and online surveys were conducted at seven time points. Primary analyses cover waves 4 (May/June 2020), 5 (July/August 2020), 6 (October 2020) and 7 (February 2021), including a period of increased restrictions in the UK. Mental health indicators were suicidal ideation, self-harm, suicide attempt, depression, anxiety, defeat, entrapment, loneliness and well-being.

**Results:**

A total of 2691 (87.5% of wave 1) individuals participated in at least one survey between waves 4 and 7. Depressive symptoms and loneliness increased from October 2020 to February 2021. Defeat and entrapment increased from July/August 2020 to October 2020, and remained elevated in February 2021. Well-being decreased from July/August 2020 to October 2020. Anxiety symptoms and suicidal ideation did not change. Young adults, women, those who were socially disadvantaged and those with a pre-existing mental health condition reported worse mental health.

**Conclusions:**

The mental health and well-being of the UK population deteriorated from July/August 2020 to October 2020 and February 2021, which coincided with the second wave of COVID-19. Suicidal thoughts did not decrease significantly, suggesting a need for continued vigilance as we recover from the pandemic.

The COVID-19 pandemic is one of the biggest global health crises that the world has faced, and the longer-term impact of the pandemic on the mental health and well-being of people globally remains unclear.^1,2^ However, it is evident that its effects stretch beyond those who have been infected with the COVID-19 virus itself (SARS-CoV-2), as the public health decisions taken to mitigate its spread have led to restrictions on movement and social interactions, and the closing of non-essential services. Within the UK, the first national lockdown commenced on 23 March 2020, with two subsequent lockdowns commencing on 31 October 2020 and 6 January 2021, although this varied across the nations and regions of the UK (for further information on the UK's COVID-19 response, please see: https://www.gov.uk/guidance/covid-19-coronavirus-restrictions-what-you-can-and-cannot-do?priority-taxon=774cee22-d896-44c1-a611-e3109cce8eae).

Findings from waves 1 to 3 of the UK COVID-19 Mental Health and Wellbeing study (UK COVID-MH^3^), covering a 6-week period at the start of the first UK lockdown (31 March 2020 to 11 May 2020), suggested that although mental health appeared to be adversely affected, some mental well-being indicators improved in the short term. For example, although still high, anxiety symptoms, defeat and entrapment decreased, whereas depressive symptoms and loneliness stayed the same. However, the proportion of those reporting suicidal thoughts increased over the waves, rising from 8.2% in wave 1 to 9.8% in wave 3. This finding was concerning, although there has yet to be consistent evidence of an increase in suicide rates linked to the pandemic in the UK or globally.^4^ The findings from waves 1 to 3 also suggested that those of a younger age, women, individuals with a pre-existing mental health condition and those from a lower socioeconomic group (SEG) reported worse mental health at each wave. However, we do not know whether these effects extended beyond the initial lockdown, in particular, whether the second wave of COVID-19 (which started in September 2020 and lasted until April 2021^5^) and the further lockdown restrictions in the UK had an impact on people's mental health and well-being. Therefore, this paper outlines the mental health and well-being of the participants in the UK COVID-MH study, spanning wave 4 (May/June 2020) through to wave 7 (February 2021), specifically focusing on the at-risk subgroups.

Comparing pre-pandemic levels of mental health, a recent systematic review has suggested that after a significant overall increase in mental health symptoms during March/April 2020, there was a decline in these rates into May/June 2020.^6^ This suggests that mental health improved over time, specifically during a time when restrictions were eased. Indeed, evidence from another review suggests that lockdowns have a negative psychological effect, although this is not homogenous.^7^ What is unclear is whether the restrictions, including a subsequent lockdown, experienced in the UK from October 2020 to March 2021 had a further impact on mental health and well-being. Evidence from the COVID-19 Social Study suggests rises in anxiety and depression levels since the end of the summer 2020,^8^ but overall there appears to be a dearth of research into the psychological impact of the subsequent lockdowns. Therefore, in the current study, as wave 6 (October 2020) and wave 7 (February 2021) are conducted during times of increased restrictions, we can more thoroughly examine whether these are associated with poorer mental health.

## Aims

The current study reports the mental health and well-being of adults from across the UK during the COVID-19 pandemic, specifically data from seven time points, spanning 12 months: wave 1 (March/April 2020), wave 2 (April 2020), wave 3 (April/May 2020), wave 4 (May/June 2020), wave 5 (July/August 2020), wave 6 (October 2020) and wave 7 (February 2021) of the UK COVID-MH study. As data from wave 1 (March/April 2020) to wave 3 (May 2020) of the study have been published previously,^3^ the focus of the analysis in this paper is on changes from wave 4 (May/June 2020) to wave 7 (February 2021), specifically investigating changes in mental well-being during the second wave of COVID-19 and increased restrictions in autumn 2020 and winter 2021. Mental health and well-being outcomes included depressive symptoms, anxiety symptoms, suicidal ideation, defeat, entrapment, loneliness and mental well-being. In addition to reporting changes in outcomes over the waves, we investigated whether outcomes varied by demographic characteristics (age, gender), SEG or the presence of a pre-existing mental health condition.

## Method

### Study design, setting and participant recruitment

Participant recruitment for the UK COVID-MH study was conducted by Taylor McKenzie, a social research company. A non-probability quota sample of adults (aged 18 years or older) from across the UK was recruited in March 2020. A quota sampling methodology was employed (see O'Connor et al^3^ for more detail) to recruit a stratified sample during the early phase of lockdown, with quotas based on age, gender, SEG and UK region (12 regions). This study had a longitudinal design that included at least six follow-ups. Because of COVID-19 restrictions, data were collected online in all waves.

At wave 1 (31 March to 9 April 2020), participants of an online UK panel called Panelbase.net were invited by email to participate in an online survey tracking the health and well-being of people in the UK. Eligibility for the study was assessed with demographic questions based on the quotas. Eligible participants then provided informed consent online, and completed a range of psychological and social measures. Participants completed two further waves within the following 6 weeks, wave 2 (10–27 April 2020) and wave 3 (28 April to 11 May 2020), reported previously.^3^ The current paper primarily reports analysis from wave 4 (27 May to 15 June 2020), wave 5 (17 July to 17 August 2020), wave 6 (1 October to 4 November 2020) and wave 7 (4 February to 2 March 2021). Findings reported here are for depressive symptoms, anxiety symptoms, suicidal ideation, self-harm history, defeat, entrapment, loneliness and well-being.

Those who took part in wave 1 were then invited by email to take part in the follow-up waves, with the exception of waves 4 and 5. Because of a technical error, only those who had completed the previous wave (wave 3) were asked to take part in waves 4 and 5, but all respondents were once again invited to complete waves 6 and 7; 15.4% (*n* = 473) of the wave 1 sample did not take part in wave 3, and 24.3% (*n* = 115) of that group completed wave 6 when invited. Those who did not complete wave 6 when re-invited (*n* = 358; 75.7% of those who did not complete wave 3) were younger (*χ*^2^ = 7.44, *P* = 0.024, *φ* = 0.13) and reported higher anxiety symptoms (*χ*^2^ = 5.57, *P* = 0.018, *φ* = −0.11), although there were no differences between gender, SEG, pre-existing mental health condition, depressive symptoms or suicidal ideation. Therefore, as the effect sizes of these differences were small, we have retained these participants in the analyses. Respondents were included in the analyses if they had taken part in at least one wave of data collection from wave 4 to wave 7. The sample characteristics are presented in Table 1 (*n* = 2691).

**Table 1:** Demographic characteristics of the waves 4–7 sample (*n* = 2691) compared with the wave 1 sample (*n* = 3077)

Characteristic	Wave 1 sample, *n* = 3077	Waves 4–7 sample, *n* = 2691
	*n* (%)	*n* (%)
Gender at birth^a^		
Men	1381 (44.9)	1251 (46.5)
Women	1692 (55.1)	1438 (53.5)
Age		
18–29 years	847 (27.5)	638 (23.7)
30–59 years	1636 (53.2)	1484 (55.1)
≥60 years	594 (19.3)	569 (21.1)
Ethnicity		
White	2777 (90.5)	2452 (91.1)
Asian	162 (5.3)	135 (5.0)
Black	68 (2.2)	54 (2.0)
Mixed	52 (1.7)	35 (1.3)
Other/unknown	18 (0.6)	15 (0.5)
Region of UK		
South England	1284 (41.7)	1102 (41.0)
Midlands	401 (13.0)	362 (13.5)
North England	845 (27.5)	740 (27.5)
Scotland	350 (11.4)	316 (11.7)
Wales	136 (4.4)	118 (4.4)
Northern Ireland	61 (2.0)	53 (2.0)
Relationship status		
Married/living with partner	1834 (59.6)	1618 (60.1)
Single	962 (31.3)	820 (30.5)
Separated/divorced/widowed	248 (8.1)	231 (8.6)
Other/prefer not to say	33 (1.1)	22 (0.8)
Sexuality		
Heterosexual	2830 (92.0)	2492 (92.9)
Gay or bisexual	220 (7.1)	177 (6.6)
Other/prefer not to say	27 (0.9)	22 (0.8)
Employment status		
Employed	1838 (59.7)	1577 (58.6)
Unemployed	358 (11.6)	322 (12.0)
Other (retired, education, homemaker)	881 (28.6)	797 (29.4)
Socioeconomic grouping^b^		
High	1758 (57.1)	1535 (57.0)
Low	1319 (42.9)	1156 (43.0)
Tenure		
Own (including with mortgage)	1835 (59.6)	1635 (60.8)
Private rent	694 (22.6)	585 (21.7)
Council rent	463 (15.0)	404 (15.0)
Other	85 (2.8)	67 (2.5)
Pre-existing mental health condition		
No	2241 (72.8)	1996 (74.2)
Yes	836 (27.2)	695 (25.8)

a. Wave 1, *n* = 3073; waves 4–7 *n* = 2689.

b. Categories A, B and C1 indicate high socioeconomic group; categories C2, D and E indicate low socioeconomic group.

**Fig. 1:**
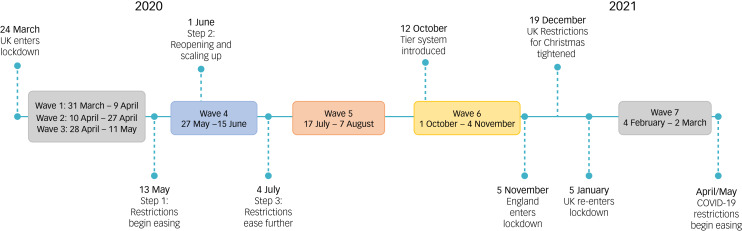
Overview of the waves of the UK COVID-19 Mental Health and Wellbeing study and key events during the COVID-19 pandemic in the UK.

Figure 1 illustrates the changes in public health restrictions in the UK occurring during each wave. The first three waves occurred within the first 6 weeks of the UK lockdown, and the subsequent four waves were roughly every 2–3 months, with the interval between waves increasing over time. During waves 1–3, the initial lockdown was underway. Wave 4 (May/June 2020) coincided with the easing of some restrictions across the UK (e.g. England on 13 May). During wave 5 (July/August 2020), restrictions had mostly been relaxed across the UK. Wave 6 (October 2020) coincided with the increasing of restrictions again across the UK, with cases of COVID-19 on the rise. During wave 7 (February 2021), there was a UK-wide lockdown. To ensure that it is clear when each wave occurred, they will be referred to by the main month and year when they occurred (e.g. wave 4, May/June 2020), where appropriate.

**Fig. 2:**
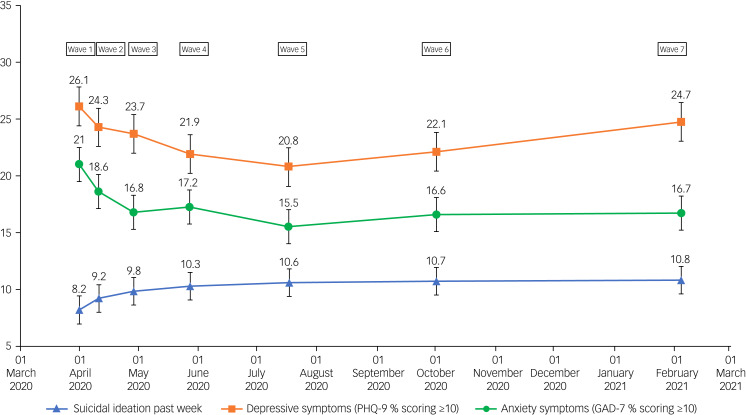
Percentages and 95% confidence intervals (error bars) of suicidal ideation, depressive symptoms and anxiety symptoms (percentage of participants scoring ≥10) over waves 1–7 of the UK COVID-19 Mental Health and Wellbeing study (*N* = 3077). GAD-7, seven-item Generalised Anxiety Disorder assessment; PHQ-9, nine-item Patient Health Questionnaire.

**Fig. 3:**
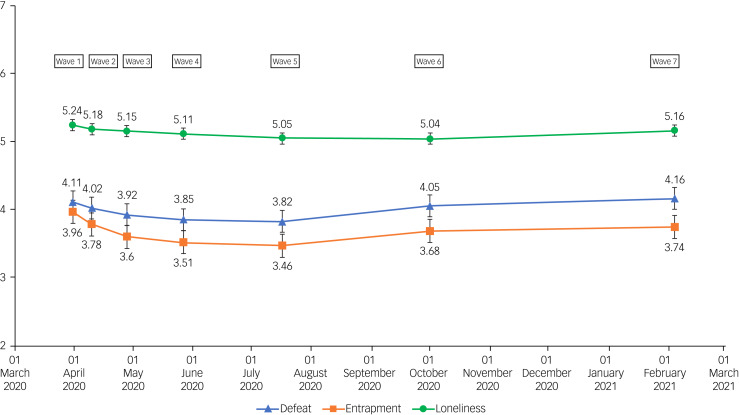
Means and 95% confidence intervals of defeat, entrapment and loneliness scores over waves 1–7 of the UK COVID-19 Mental Health and Wellbeing study (*N* = 3077).

In the interests of completeness, data for all waves are included in Table 2 and Figs. 2 and 3.

**Table 2:** Changes in primary outcome variables over waves 1–7 of the UK COVID-19 Mental Health and Wellbeing study, with odds ratios and 95% confidence intervals

	Wave 1: March/April 2020,	Wave 2: April 2020,	Wave 3: April/May 2020,	Wave 4: May/June 2020,	Wave 5: July/August 2020,	Wave 6: October 2020,	Wave 7: February 2021,	Odds ratio [95% CI], *P*-value
	*n* = 3077,	*n* = 2742,	*n* = 2604,	*n* = 2384,	*n* = 2144,	*n* = 2283,	*n* = 2224,	
	% [95% CI]	% [95% CI]	% [95% CI]	% [95% CI]	% [95% CI]	% [95% CI]	% [95% CI]	
Suicidal ideation in the past week	8.2 [7.2−9.2]	9.2 [8.1−10.3]	9.8 [8.7−10.9]	10.3 [9.1−11.6]	10.6 [9.3−12.0]	10.7 [9.4−12.1]	10.8 [9.5−12.2]	Waves 4–5^a^ = 1.05 [0.90−1.23], *P* = 0.547 Waves 5–6^b^ = 1.03 [0.88−1.20], *P* = 0.727 Waves 6–7^c^ = 1.00 [0.86−1.17], *P* = 0.959
Suicide attempts in the past week	0.1 [−0.3 to 0.5]	0.8 [0.4−1.2]	0.7 [0.3−1.1]	0.8 [0.5−1.2]	0.7 [0.4−1.1]	0.9 [0.5−1.3]	0.9 [0.5−1.3]	Not applicable
Self- harm in the past week	0.7 [0.4−1.1]	1.8 [1.3−2.3]	1.4 [1.0−1.9]	1.6 [1.1−2.1]	1.2 [0.7−4.7]	1.8 [1.3−2.4]	1.5 [1.0−2.0]	Not applicable
PHQ-9 (percentage scoring ≥10)	26.1 [24.6−27.7]	24.3 [22.7−25.9]	23.7 [22.1−25.3]	21.9 [20.3−23.7]	20.8 [13.1−22.6]	22.1 [20.4−23.8]	24.7 [22.9−26.5]	Waves 4–5^a^ = 0.98 [0.88−1.08], *P* = 0.645 Waves 5–6^b^ = 1.07 [0.96−1.19], *P* = 0.222 Waves 6–7^c^ = 1.13 [1.03−1.26], *P* = 0.015
GAD-7 (percentage scoring ≥10)	21 [19.6−22.4]	18.6 [17.1−20.1]	16.8 [15.4−18.2]	17.2 [15.7−18.7]	15.5 [14.0−17.1]	16.6 [15.1−18.2]	16.7 [15.2−18.3]	Waves 4–5^a^ = 0.93 [0.82−1.05], *P* = 0.227 Waves 5–6^b^ = 1.07 [0.95−1.21], *P* = 0.255 Waves 6–7^c^ = 1.02 [0.91−1.15], *P* = 0.745
	Mean [95% CI]	Mean [95% CI]	Mean [95% CI]	Mean [95% CI]	Mean [95% CI]	Mean [95% CI]	Mean [95% CI]	
Defeat	4.11 [4.11−4.39]	4.02 [3.87−4.17]	3.92 [3.77−4.07]	3.85 [3.69−4.01]	3.82 [3.65−3.99]	4.05 [3.88−4.22]	4.16 [3.99−4.33]	Waves 4–5^a^ = 1.04 [0.89−1.22], *P* = 0.598 Waves 5–6^b^ = 1.22 [1.04−1.43], *P* = 0.017 Waves 6–7^c^ = 1.12 [0.96−1.30], *P* = 0.150
Entrapment	3.96 [3.81−4.11]	3.78 [3.62−3.94]	3.60 [3.44−3.76]	3.51 [3.34−3.68]	3.46 [3.28−3.64]	3.68 [3.50−3.86]	3.74 [3.56–3.92]	Waves 4–5^a^ = 1.02 [0.87−1.21], *P* = 0.770 Waves 5–6^b^ = 1.21 [1.02−1.44], *P* = 0.029 Waves 6–7^c^ = 1.07 [0.90−1.26], *P* = 0.451
Loneliness	5.24 [5.17−5.31]	5.18 [5.11−5.25]	5.15 [5.08−5.22]	5.11 [5.03−5.19]	5.05 [4.97−5.13]	5.04 [4.96−5.12]	5.16 [5.08−5.24]	Waves 4–5^a^ = 0.97 [0.91−1.05], *P* = 0.482 Waves 5–6^b^ = 1.00 [0.93−1.07], *P* = 0.904 Waves 6–7^c^ = 1.11 [1.03−1.19], *P* = 0.008
Well-being	22.27 [22.05−22.49]	22.64 [22.41−22.87]	22.92 [22.68−23.16]	23.33 [23.10−23.59]	23.45 [23.18−23.72]	23.15 [22.89−23.42]	23.42 [23.15−23.69]	Waves 4–5^a^ = 1.03 [0.81−1.32], *P* = 0.819 Waves 5–6^b^ = 0.75 [0.58−0.96], *P* = 0.021 Waves 6–7^c^ = 1.22 [0.95−1.58], *P* = 0.124

Data are provided for all waves but analysis is for waves 4–7 only; analysis for waves 1–3 can be found in O'Connor et al.^3^ PHQ-9, nine-item Patient Health Questionnaire; GAD-7, seven-item Generalised Anxiety Disorder assessment.

a. Reference group wave 4.

b. Reference group wave 5.

c. Reference group wave 6.

The authors assert that all procedures contributing to this work comply with the ethical standards of the relevant national and institutional committees on human experimentation and with the Helsinki Declaration of 1975, as revised in 2008. All procedures involving human participants were approved by the University of Glasgow's Medical, Veterinary and Life Sciences Ethics Committee (approval number: 200190146). The study was preregistered at AsPredicted.org (identifier: 41910). Study participants were compensated approximately £1.50 for the completion of each survey, and were entered into prize draws. Details of mental health support organisations were made available to participants.

### Measures

All suicide-related items were assessed within the time frame of the past week. Suicidal ideation was measured with ‘How often have you thought about taking your life in the past week? (‘one day’, ‘several days’, ‘more than half the days’, ‘nearly every day’, ‘never’, ‘I would rather not answer’)’; ‘one day’ to ‘nearly every day’ were coded as yes, and ‘never’ was coded as no. Suicide attempt and self-harm were measured with ‘In the past week, have you made an attempt to take your life, e.g. by taking an overdose of tablets or in some other way?’ and ‘In the past week, have you ever deliberately harmed yourself in any way but not with the intention of killing yourself?’ (‘yes’, ‘no, ‘I would rather not answer’).

Depressive symptoms were assessed with the nine-item Patient Health Questionnaire (PHQ-9^9^), and anxiety symptoms were assessed with the seven-item Generalised Anxiety Disorder (GAD-7) scale.^10^ Both tools ask how often symptoms have bothered the respondents in the previous 2 weeks. A score of ≥10 on both measures is suggested to indicate moderate levels (or more) of depression and anxiety, and this cut-off is used in the current study.^10,11^

Feelings of defeat (perceived failure and loss of rank) were assessed with the four-item defeat subscale of the Short Defeat and Entrapment Scale by Griffiths et al.^12^ Perceptions of entrapment (feeling trapped by thoughts and feelings or circumstances) were measured with the Entrapment Scale Short-Form.^13^ Mental well-being was assessed with the seven-item Short Warwick–Edinburgh Mental Well-Being Scale.^14^ Loneliness was measured with the Three-Item Loneliness Scale, developed from the Revised UCLA Loneliness Scale.^15^ Percentages are reported for all binary outcome variables (suicidal ideation, depressive symptoms and anxiety symptoms) and mean scores are reported for all continuous outcome variables (defeat, entrapment, well-being and loneliness).

SEG was assessed according to the National Readership Survey social grade:^16^ high (categories A, B and C1) versus low (categories C2, D and E). Participants were asked whether they had any long-standing physical or mental impairment, illness or disability, and this was coded to create a dichotomous variable for presence or absence of a pre-existing mental health condition. Specifically, participants were asked to select their mental or physical impairment from a list of options, which included mental health conditions, neurodivergent disorders and alcohol and drug problems.

### Statistical analysis

Analysis of data from waves 4–7 was consistent with data from the previous waves 1–3 where possible, to aid comparison. All analyses were conducted with Stata MP 16 (Windows), using an imputed data-set of the 2691 participants who completed at least one survey from waves 4 to 7.

We used multiple imputation to generate 50 data-sets for each outcome variable. Multiple imputation is a statistical technique whereby an imputation represents one set of plausible values for missing data, and the imputation model for deriving these imputations includes predictors relevant to the missing-data mechanism. In analysis, the results from the multiple imputations are pooled into a single result.^17^ The binary variables (suicidal ideation, depressive symptoms, anxiety symptoms) were imputed with logistic regression (mi impute logit), and continuous variables (defeat, entrapment, loneliness, well-being) were imputed with linear regression (mi impute regress). The imputation model included age, gender, SEG, history of mental health condition and the region of residence in the UK.

Participants (*n* = 2691) were included in the main analysis if they completed at least one survey from waves 4 to 7; therefore, there was missing data at each wave, and this varied across waves. Across these four waves, when totalled up, each variable had approximately 16.1% (*n* = 1729/10 764) missing cases (percentage missing at each wave: *n* = 307, 12.9% at wave 4; *n* = 547, 25.5% at wave 5; *n* = 4.8, 15.2% at wave 6; *n* = 467, 17.4% at wave 7). Suicidal ideation had 2.7% (*n* = 299) more missing cases (18.8%, *n* = 2028/10 764), as an option for ‘would rather not answer’ was included with this item (percentage answering ‘would rather not answer’ at each wave: 2.4% at wave 4, 2.4% at wave 5, 3.2% at wave 6, 3.0% at wave 7). Full details of missing data (‘would rather not answer’) and the frequencies of respondents who reported suicidal ideation, suicide attempt and self-harm are included in the supplementary materials (Supplementary Table 1 available at https://doi.org/10.1192/bjo.2022.58). For suicide attempts and self-harm, often there was more missingness than the prevalence of that outcome, but no inferential statistics are applied to these outcomes.

Multiple imputation generalised estimating equations (GEE) models were then constructed to test the changes in the variables across waves 4–7 for the whole sample. This approach is suitable for multilevel longitudinal panel data.^18^ In a sensitivity check, all analyses were conducted with and without multiple imputation, and a similar pattern was found for both, with the multiple imputation analysis more conservative and reported here. GEE models apply a multilevel method,. Binomial logit modelling was conducted for each of the binary outcome variables (suicidal ideation, depressive symptoms and anxiety symptoms), and linear Gaussian identity modelling was conducted for the continuous outcome variables (defeat, entrapment, loneliness and well-being).

To test for subgroup differences, additional GEE models were applied to the outcome variables, investigating differences in age (18–29, 30–59, ≥60 years), gender (men, women), SEG (higher, lower) and a pre-existing mental health problem (no, yes). Unlike the waves 1–3 analysis, ethnicity was not included because of reduced numbers. Interactions between the subgroups and changes in the outcomes over the waves were then tested, with significant findings only reported in the results.

The region of the UK in which a participant lived (South England, Midlands, North England, Scotland, Wales and Northern Ireland) was controlled for in each analysis. The temporal covariation was modelled with an unstructured correlation matrix, as the pattern of associations was neither fully exchangeable nor had a first-order autoregressive structure. Additionally, because of the large number of analyses, a false discovery rate procedure^19^ was applied to the between, within and interaction *P*-values for all analyses. The false discovery rate procedure places all *P*-values in ascending order and assigns ranks (e.g. smallest is ranked 1), then the Benjamini–Hochberg critical value for each *P*-value is calculated with the formula (*i*/*m*)*Q* (with *i* being the individual *P*-value's rank, *m* being the total number of tests and *Q* being the false discovery rate (0.05)), and the largest *P*-value that is smaller than its critical value (*P* < (*i*/*m*)*Q*) is significant, along with all smaller *P*-values. This method adjusts for type 1 errors in null hypothesis testing when conducting multiple comparisons, as it controls for the expected proportion of ‘discoveries’ that are false.^20^

## Results

At wave 1 (March/April 2020), 3077 participants took part in the initial wave of data collection. The main analyses in this paper focus on data from waves 4 (May/June 2020) to 7 (February 2021), and this sample consisted of 2691 participants who took part in at least one survey between May 2020 and February 2021, which was 87.5% of the original sample (Table 1). Those who dropped out of the study were younger (*χ*^2^ = 167.02, *P* < 0.001, *φ* = 0.23) and more likely to be female (*χ*^2^ = 21.80, *P* < 0.001, *φ* = 0.08) and score higher on wave 1 depressive (*χ*^2^ = 34.31, *P* < 0.001, *φ* = −0.11) and anxiety (*χ*^2^ = 46.95, *P* < 0.001, *φ* = −0.12) symptoms. The sample was made up of 53.5% (*n* = 1438) women, 55.1% (*n* = 1484) were aged between 30 and 59 years and 91.1% (*n* = 2452) were from a White background. Two-thirds (60.1%) were married or living with a partner, 92.9% identified as heterosexual and over half (58.6%) were employed.

### Mental health outcomes from wave 1 (March/April 2020) to wave 7 (February 2021)

Table 2 reports the mental health outcomes (percentages and means) for all study participants from March/April 2020 (wave 1) to February 2021 (wave 7). Figure 2 displays the changes in rates of suicidal ideation, depressive symptoms and anxiety symptoms for the whole sample at each study wave, and Figure 3 displays the changes in in levels of defeat, entrapment and loneliness for the whole sample at each study wave (a figure illustrating levels of well-being across the waves is included in the supplementary materials; see Supplementary Figure 1).

Visual trends across the waves suggest that mental health was poorer at the start of the pandemic, with rates of depressive symptoms, anxiety symptoms, entrapment and loneliness being the highest in March/April 2020 (wave 1), compared with other waves. This was followed by a decline in symptoms of poor mental health throughout the spring and summer of 2020, as documented previously.^3^ This appears to be followed by a gradual increase in symptoms of poor mental health from July/August 2020 (wave 5) to October 2020 (wave 6) and February 2021 (wave 7). The main exception to these trends is rates of suicidal ideation, which increased significantly from March/April 2020 (wave 1) to April/May 2020 (wave 3),^3^ and then remained at a similar level through May/June 2020 (wave 4) to February 2021 (wave 7).

### Statistical changes from wave 4 (May/June 2020) to wave 7 (February 2021)

Table 2 reports the statistical changes from May/June 2020 (wave 4) to February 2021 (wave 7). There were no significant changes in rates of suicidal ideation in the past week between waves, with 10.3% of the sample reporting suicidal ideation in May/June 2020 (wave 4) and 10.8% in February 2021 (wave 7). Rates of suicide attempts (range: 0.7–0.9%) and self-harm (range: 1.2–1.8%) in the past week remained low.

There were no significant changes in rates of depressive symptoms between May/June 2020 (wave 4, 21.9%) and October 2020 (wave 6, 22.1%), but there was a significant increase from October 2020 to February 2021 (wave 7, 24.7%). Depressive symptoms in February 2021 (wave 7) were higher than May/June 2020 (wave 4: odds ratio 1.18, 95% CI 1.06–1.32, *P* = 0.003), July/August 2020 (wave 5: odds ratio 1.21, 95% CI 1.09–1.36, *P* = 0.001) and October 2020 (wave 6: odds ratio 1.02, 95% CI 1.03–1.26, *P* = 0.015). Anxiety symptoms did not change significantly over the waves, with 17.2% meeting the cut-off for moderate anxiety in May/June 2020 (wave 4), and 16.7% meeting the cut-off in February 2021 (wave 7).

Mean scores for defeat did not change between May/June 2020 (wave 4, 3.85) and July/August 2020 (wave 5, 3.82). Defeat significantly increased from July/August 2020 (wave 5, 3.82) to October 2020 (wave 6, 4.05; odds ratio 1.22, 95% CI 1.04–1.43, *P* = 0.017), and from July/August 2020 (wave 5, 3.82) to February 2021 (wave 7, 4.16; odds ratio 1.36, 95% CI 1.16–1.60, *P* < 0.001). Similarly, mean entrapment scores did not change significantly from May/June 2020 (wave 4, 3.51) to July/August 2020 (wave 5, 3.46). Entrapment increased from July/August 2020 (wave 5) to October 2020 (wave 6, 3.68; odds ratio 1.21, 95% CI 1.02–1.44, *P* = 0.029) and from July/August 2020 (wave 5) to February 2021 (wave 7, 3.74; odds ratio 1.29, 95% CI 1.08–1.54, *P* = 0.005). The change from October 2020 (wave 6, 3.68) to February 2021 (wave 7, 4.16) was not significant.

Loneliness mean scores did not change significantly in May/June 2020 (wave 4, 5.11), July/August 2020 (wave 5, 5.05) or October 2020 (wave 6, 5.04). There was a significant increase in loneliness from July/August 2020 (wave 5, 5.05) to February 2021 (wave 7, 5.16; odds ratio 1.10, 95% CI 1.02–1.19, *P* = 0.019) and from October 2020 (wave 6, 5.04) to February 2021 (wave 7, 5.16; odds ratio 1.11, 95% CI 1.03–1.19, *P* = 0.008). Mental well-being scores decreased from July/August 2020 (wave 5, 23.45) to October 2020 (wave 6, 23.45; odds ratio 0.75, 95% CI 0.58–0.96, *P* = 0.02).

### Subgroup mental health outcomes: gender, age, SEG and pre-existing mental health condition

Consistent with the previous findings, particular subgroups reported worse overall mental health at each wave. All group comparison findings are included within Table 3, with the group differences reported in Supplementary Tables 1–4 in the supplementary materials.

**Table 3:** Table of generalised estimating equations model output for subgroup comparisons for each variable

	Age,^a^ odds ratio [95% CI], *P*-value	Age,^b^ odds ratio [95% CI], *P*-value	Gender,^c^ odds ratio [95% CI], *P*-value	Mental health,^d^ odds ratio [95% CI], *P*-value	Socioeconomic group,^e^ odds ratio [95% CI], *P-*value
Suicidal ideation	0.56 [0.42–0.74], *P* < 0.001	0.27 [0.15–0.46], *P* < 0.001	0.87 [0.66–1.15], *P* = 0.323	3.01 [2.29–3.97], *P* < 0.001	1.17 [0.90–1.53], *P* = 0.232
Depressive symptoms	0.63 [0.51–0.79], *P* < 0.001	0.38 [0.28–0.53], *P* < 0.001	0.55 [0.45–0.67], *P* < 0.001	5.11 [4.16–6.29], *P* < 0.001	1.37 [1.13–1.66], *P* < 0.001
Anxiety symptoms	0.62 [0.49–0.78], *P* < 0.001	0.36 [0.25–0.52], *P* < 0.001	0.53 [0.43–0.67], *P* < 0.001	4.85 [3.87–6.07], *P* < 0.001	1.32 [1.06–0.62], *P* = 0.010
Defeat	0.45 [0.30–0.67], *P* < 0.001	0.17 [0.11–0.25], *P* < 0.001	0.29 [0.21–0.40], *P* < 0.001	36.51 [26.05–51.18], *P* < 0.001	2.37 [1.70–3.30], *P* < 0.001
Entrapment	0.09 [0.36–0.82], *P* = 0.003	0.16 [0.11–0.25], *P* < 0.001	0.34 [0.24–0.48], *P* < 0.001	44.41 [30.92–63.80], *P* < 0.001	2.10 [1.48–2.95], *P* < 0.001
Loneliness	0.68 [0.83–1.31], *P* < 0.001	0.48 [0.40–0.58], *P* < 0.001	0.54 [0.46–0.63], *P* < 0.001	3.29 [2.78–3.89], *P* < 0.001	1.33 [1.14–1.55], *P* < 0.001
Well-being	4.05 [2.17–7.53], *P* < 0.001	28.18 [14.82–53.58], *P* < 0.001	3.84 [2.30–6.42], *P* < 0.001	0.01 [0.01–0.02], *P* < 0.001	0.44 [0.26–0.73], *P* = 0.002

a. 18–29 years compared with 30–59 years.

b. 30–59 years compared with ≥60 years.

c. Women compared with men.

d. No pre-existing mental health condition compared with having a pre-existing mental health condition.

e. High socioeconomic group compared with low socioeconomic group.

From May/June 2020 (wave 4) to February 2021 (wave 7), young adults (aged 18–29 years) reported higher suicidal ideation, depressive symptoms, anxiety symptoms, defeat, entrapment and loneliness, along with lower well-being, compared with age groups 30–59 and ≥60 years. Additionally, those aged 30–59 years reported higher rates of suicidal ideation, depressive symptoms, anxiety symptoms, defeat, entrapment and loneliness compared with those aged ≥60 years. Women also reported higher rates of mental ill health than men on all outcomes, except for suicidal ideation, which was not statistically different (Table 3).

Respondents who reported having a pre-existing mental health condition had consistently higher rates of suicidal ideation, depressive symptoms, anxiety symptoms, defeat, entrapment and loneliness compared with those with no pre-existing mental health condition, and similarly, well-being scores were lower for this group. Those from the lower SEG also reported higher depressive symptoms, anxiety symptoms, defeat, entrapment and loneliness, and lower well-being, but did not report any differences in rates of suicidal ideation.

### Changes across the waves by subgroup

For each of the subgroups, there were no significant differences in changes over time in anxiety symptoms, defeat, loneliness or well-being. Although 20.2% of young adults reported suicidal ideation by February 2021 (wave 7), compared with 17.1% in May/June 2021 (wave 4), this increase was not significant in the multiple imputation GEE analysis (odds ratio 0.80, 95% CI 0.57–1.12, *P* = 0.203). It is worth noting, however, that suicidal ideation at wave 7 was the highest rate reported by young adults across the seven waves.

From October 2020 (wave 6) to February 2021 (wave 7), depressive symptoms increased for those with no pre-existing mental health condition (wave 6, 13.4%; wave 7, 17.3%; odds ratio 0.76, 95% CI −0.60 to 0.96, *P* = 0.021). Compared with those aged 30–59 years, entrapment increased from May/June 2021 (wave 4, 4.32) to October 2020 (wave 6, 4.47) for those aged 18–29 years (odds ratio 0.66, 95% CI 0.44–0.999, *P* = 0.05). Entrapment also increased for women from May/June 2021 (wave 4, 4.00) to October 2020 (wave 6, 4.34) compared with men (odds ratio 0.72, 95% CI 0.53–0.98, *P* = 0.038).

## Discussion

The aim of the current study was to examine the mental health and well-being of a sample of adults from across the UK, during the COVID-19 pandemic. Specifically, data are presented that cover a period of almost 12 months, during which there was an initial lockdown and then gradual easing of restrictions, followed by the second wave of COVID-19, which resulted in two national lockdowns. Data from the early phase of the pandemic (March/April 2020 to May 2020, waves 1–3) suggest that mental health improved as restrictions eased.^3^ Evidence suggests that from May/June 2020 (wave 4) to July/August 2020 (wave 5), when restrictions in the UK continued to ease, there were no significant changes in the mental health indicators. As most mental health indictors had improved by late April/May 2020, this suggests that there was not much fluctuation in mental health outcomes during the late spring and summer months. This is consistent with findings from a systematic review indicating an overall improvement in rates of mental health symptoms from March/April 2020 to May/June 2020.^6^ The present study further suggests that when restrictions increased, from July/August 2020 to October 2020, and again to February 2021, with the implementation of a national lockdown, there was clear evidence of a worsening of mental health. Specifically, depressive symptoms and loneliness increased from October 2020 (wave 6) to February 2021 (wave 7), and feelings of defeat and entrapment increased from July/August 2020 (wave 5) to October 2020 (wave 6), and this increase persisted to February 2021 (wave 7). Mental well-being decreased from July/August 2020 (wave 5) to October 2020 (wave 6). Rates of suicidal ideation and anxiety symptoms did not change over these waves, although there had been a rise in suicidal ideation in the first 6 weeks of the pandemic.

Several subgroups reported worse mental health outcomes. Specifically, young adults were higher on all mental health indicators, including higher rates of suicidal ideation, depressive and anxiety symptoms at each wave, compared with those who were aged 30–59 years, who in turn reported higher rates than those who were aged ≥60 years. By February 2021 (wave 7), a fifth (20.2%) of young adults reported experiencing suicidal ideation in the previous week, and although this increase was not statistically significant (from 17.1% at May/June 2020, wave 4), it is concerning as it is the highest rate reported across the seven waves. Additionally, young adults reported an increase in feelings of entrapment from May/June 2020 (wave 4) to October 2020 (wave 6), which is a recognised risk factor in the development of suicidal ideation.^21^ Women reported worse mental health outcomes than men across most indicators, except for suicidal ideation; and entrapment also increased for women from May/June 2020 (wave 4) to October 2020 (wave 6). Those from a more disadvantaged background were also higher on all mental ill health indicators (except for suicidal ideation) when compared with those from more advantaged backgrounds. Across every indicator, those with a pre-existing mental health condition reported the worst mental health outcomes; however, interestingly, rates of depressive symptoms increased from October 2020 (wave 6) to February 2021 (wave 7) in respondents with no pre-existing mental health condition.

### Implications

From May/June 2020 to February 2021, approximately one in ten respondents reported experiencing suicidal ideation in the past week (10.3% at May/June 2020, wave 4; 10.8% at February 2021, wave 7). Although suicidal thinking did not increase over this time frame, it still represents an increase from 8.2% at March/April 2020 (wave 1),^3^ and suggests that rates of suicidal ideation did not improve with the changing of restrictions. A recent meta-analysis proposes that rates of suicidal thoughts and behaviours have been higher during the COVID-19 pandemic compared with pre-pandemic levels.^22^ Thus far, there is little evidence that this risk has translated into suicide deaths,^4^ although continued monitoring and vigilance is required.

Findings indicate that overall mental health deteriorated during the second wave of COVID-19, and this coincided with a period of increased government restrictions. Specifically, depressive symptoms increased, with nearly a quarter (24.7%) of respondents meeting the cut-off for moderate depressive symptoms in February 2021 (wave 7). In contrast, anxiety symptoms did not fluctuate significantly over the waves, with around one sixth (e.g. 16.7% in February 2021) meeting the cut-off for moderate anxiety from May/June 2020 (wave 4) to February 2021 (wave 7). The COVID-19 Social Study, a longitudinal UK study conducted by the University College London,^23^ found anxiety and depressive symptoms decreased in the initial period after lockdown as people adjusted to the measures, which aligns with the findings from waves 1–3 of the current study.^3^ The findings here suggest that subsequent lockdowns have had a further impact on people's mental well-being, leading to an increase in depressive symptoms, loneliness, defeat and entrapment, and a decrease in well-being. Particular subgroups maybe at higher risk, including younger people and women, with these higher risk subgroups identified in the COVID-19 Social Study investigating trajectories of depression and anxiety across the pandemic.^24^ Interestingly, respondents with no pre-existing mental health condition reported an increase in depressive symptoms from October 2020 (wave 6) to February 2021 (wave 7), suggesting that those who were previously mentally healthy may be increasingly affected.

Feelings of defeat and entrapment also increased, with young adults and women in particular reporting an increase in entrapment. These factors are key components of the integrated motivational–volitional model of suicidal behaviour,^25^ with evidence suggesting that they are instrumental to the emergence of suicidal thinking.^26^ Although suicidal ideation did not increase further, as established precursors for suicidal thinking have increased, care is needed to continue to monitor for suicide risk. Additionally, loneliness increased over time, potentially because of the social restrictions put in place during the lockdowns. This is concerning as loneliness is also a risk factor for future suicidal thoughts and behaviour, particularly for younger people and women,^27^ and therefore public health interventions may be needed to buffer the impact that loneliness may have in the future.

### Limitations

Similar to other survey research, there are several limitations to this study design. The recruitment method excluded those who do not have access to digital means, and therefore may have led to some selection bias. The measures are all self-reported, and do not allow for the monitoring of psychiatric disorders. Although the sample at wave 1 was nationally representative, those who dropped out at subsequent waves were younger, female and scored more poorly on most mental health indicators; therefore, the current results may provide an underestimation of the true effects. It should be noted that analyses used here to test the statistical significance of changes over the waves and differences between subgroups (such as *P*-values and 95% confidence intervals) assume the use of probability samples, whereas we have used a quota sample. Further research could use qualitative methods to enhance understanding of the mechanisms behind these changes in mental well-being, helping to mitigate the adverse impact on mental health.

In conclusion, this study provides evidence that after an initial improvement in the mental health and well-being of the UK population from March 2020 to May 2020, there was a notable deterioration from July/August 2020 to February 2021, a period that coincided with a second wave of COVID-19 and a national lockdown. Young adults, women, those who are more socially disadvantaged and individuals with a pre-existing mental health condition consistently reported the worst mental health outcomes. It is essential to continue to monitor the mental health and well-being of the UK population, and to put in place robust public health actions to mitigate the impact on longer-term mental health and well-being.

## Data Availability

The data that support the findings of this study are available from the corresponding author, K.W., upon reasonable request.

## Supplementary material

For supplementary material accompanying this paper visit https://doi.org/10.1192/bjo.2022.58. click here to view supplementary material
