# Supplementary material for: Mental health and well-being during the second wave of COVID-19: longitudinal analyses of the UK COVID-19 Mental Health and Wellbeing study (UK COVID-MH)
Source: BJPsych Open. 2022 Jun 1;8(4):e103. doi: 10.1192/bjo.2022.58 (PMC9171032; doi:10.1192/bjo.2022.58)

**Supplementary Tables**

**Table S1: Participant missing data (‘Would rather not answer’) compared to prevalence of outcome from wave 1 (March/April 2020) to wave 7 (February 2021)**

|  | **Wave 1: March/April 2020** | | **Wave 2: April 2020** | | **Wave 3: April/May 2020** | | **Wave 4: May/June 2020** | | **Wave 5: July/August 2020** | | **Wave 6: October 2020** | | **Wave 7: February 2021** | | |
| --- | --- | --- | --- | --- | --- | --- | --- | --- | --- | --- | --- | --- | --- | --- | --- |
|  | Missing N (%) | Prevalence N (%) | Missing N (%) | Prevalence N (%) | Missing N (%) | Prevalence N (%) | Missing N (%) | Prevalence N (%) | Missing N (%) | Prevalence N (%) | Missing N (%) | Prevalence N (%) | Missing N (%) | Prevalence N (%) |  |
| Suicidal ideation | 93 (3.0) | 245 (8.2) | 91 (3.3) | 245 (9.2) | 71 (2.7) | 248 (9.8) | 67 (2.8) | 239 (10.3) | 67 (3.1) | 220 (10.6) | 21 (1.0) | 235 (10.7) | 80 (3.6) | 231 (10.8) |  |
| Suicide attempts | 71 (2.3) | 4 (0.1) | 36 (1.3) | 22 (0.8) | 32 (1.2) | 17 (0.7) | 36 (1.5) | 19 (0.8) | 29 (1.4) | 15 (0.7) | 36 (1.6) | 20 (0.9) | 26 (1.2) | 20 (0.9) |  |
| Self- harm | 64 (2.1) | 23 (0.7) | 39 (1.4) | 19 (1.8) | 33 (1.3) | 35 (1.4) | 39 (1.6) | 37 (1.6) | 24 (1.1) | 25 (1.2) | 36 (1.2) | 41 (1.8) | 25 (1.1) | 32 (1.4) |  |

**Table S2. Recent suicidal history, depressive symptoms and anxiety symptoms by age group and sex**

|  | **Wave 4: May/June 2020 (n=2384)** | | | **Wave 5: July/August 2020 (n=2144)** | | | **Wave 6: October 2020 (n=2283)** | | | **Wave 7: February 2021 (n=2224)** | | |
| --- | --- | --- | --- | --- | --- | --- | --- | --- | --- | --- | --- | --- |
|  | **18- 29 years** | **30- 59 years** | **60+ years** | **18- 29 years** | **30- 59 years** | **60+ years** | **18- 29 years** | **30- 59 years** | **60+ years** | **18- 29 years** | **30- 59 years** | **60+ years** |
|  | % [95% CI] | % [95% CI] | % [95% CI] | % [95% CI] | % [95% CI] | % [95% CI] | % [95% CI] | % [95% CI] | % [95% CI] | % [95% CI] | % [95% CI] | % [95% CI] |
| Men | ***n=141*** | ***n=651*** | ***n=325*** | ***n=117*** | ***n=601*** | ***n=308*** | ***n=129*** | ***n=635*** | ***n=329*** | ***n=120*** | ***n=621*** | ***n=322*** |
| Suicidal ideation^a^ | 17.4 [11.1-23.7] | 11.0 [8.6-13.4] | 3.4 [1.4-5.4] | 18.8 [11.7-25.9] | 11.8 [9.2-14.4] | 4.2 [2.0-6.4] | 13.9 [7.9-19.9] | 12.3 [9.8-14.9] | 4.3 [2.1-6.5] | 20.2 [13.0-27.4] | 13.1 [10.5-15.8] | 3.8 [1.7-5.9] |
| Suicide attempt^a^ | 0.7 [-0.7-2.1] | 0.8 [0.1-1.5] | 0.0 [0-0] | 0.9 [-0.8-2.6] | 0.7 [0.03-1.37] | 0.3 [-0.3-0.9] | 1.6 [-0.6-3.8] | 1.3 [0.4-2.2] | 0.0 [0-0] | 1.7 [-0.6-4.0] | 1.1 [0.3-1.9] | 0.0 [0-0] |
| Self- harm^a^ | 3.4 [0.4-6.4] | 1.5 [0.6-2.4] | 1.0 [-0.8-2.1] | 1.8 [-0.6-4.2] | 1.0 [0.2-1.8] | 0.0 [0-0] | 4.8 [1.1-8.5] | 1.8 [0.8-2.8] | 0.3 [-0.3-0.9] | 0.8 [-0.8-2.4] | 2.1 [1.0-3.2] | 0.3 [-0.3-0.9] |
| PHQ9 (% ≥ 10)^b^ | 22.0 [15.8-28.8] | 20.3 [17.2-23.4] | 6.8 [4.1-9.5] | 25.6 [17.7-33.5] | 19.3 [16.1-22.5] | 8.4 [5.3-11.5] | 23.3 [16.0-30.6] | 18.1 [15.1-21.1] | 11.2 [7.8-14.6] | 33.3 [24.9] | 20.0 [16.9-23.2] | 9.6 [6.4-12.8] |
| GAD7 (% ≥ 10)^b^ | 16.3 [10.2-22.4] | 14.6 [11.9-17.3] | 6.8 [4.1-9.5] | 17.9 [11.0-24.9] | 13.3 [10.6-16.0] | 6.5 [3.8-9.3] | 13.2 [7.4-19.0] | 13.2 [10.6-15.8] | 7.0 [4.2-9.8] | 21.7 [14.3-29.8] | 12.9 [10.3-15.5] | 8.1 [5.1-11.1] |
| Women | ***n=398*** | ***n=676*** | ***n=191*** | ***n=320*** | ***n=614*** | ***n=182*** | ***n=333*** | ***n=658*** | ***n=197*** | ***n=324*** | ***n=637*** | ***n=198*** |
| Suicidal ideation | 17.0 [13.3-20.7] | 10.1 [7.8-12.4] | 2.1 [0.1-4.1] | 17.7 [13.5-21.9] | 10.2 [7.8-12.6] | 1.6 [-0.2-3.4] | 17.8 [13.7-21.9] | 11.1 [8.7-13.5] | 1.5 [-0.2-3.2] | 20.2 [15.8-24.6] | 8.9 [6.7-11.1] | 0.5 [-0.5-1.5] |
| Suicide attempt | 1.3 [0.2-2.4] | 1.2 [0.4-2.0] | 0.0 [0-0] | 1.3 [0.1-2.5] | 0.8 [0.1-1.5] | 0.0 [0-0] | 0.9 [-0.1-1.9] | 1.1 [0.3-1.9] | 0.0 [0-0] | 1.6 [0.2-3.0] | 1.0 [2.3-1.8] | 0.0 [0-0] |
| Self- harm | 1.4 [0.3-2.6] | 1.4 [0.5-2.3] | 0.3 [-0.5-1.1] | 2.6 [0.9-4.3] | 1.5 [0.5-2.4] | 0.0 [0-0] | 3.7 [1.7-5.7] | 1.7 [0.7-2.7] | 0.0 [0-0] | 2.8 [1.0-4.6] | 1.3 [0.4-2.8] | 0.0 [0-0] |
| PHQ-9 (% ≥ 10) | 34.7 [30.0-39.4] | 25.3 [22.0-28.6] | 14.7 [9.7-19.7] | 31.3 [26.2-36.4] | 25.1 [21.7-28.5] | 10.4 [6.0-14.8] | 34.5 [29.4-39.6] | 28.4 [25.0-31.8] | 10.2 [6.0-14.4] | 38.6 [33.3-43.9] | 31.6 [28.0-35.2] | 13.1 [8.4-17.8] |
| GAD-7 (% ≥ 10) | 28.6 [24.2-33.0] | 20.9 [17.9-24.0] | 7.3 [3.6-11.0] | 24.7 [20.0-29.4] | 19.9 [16.8-23.1] | 5.5 [2.2-8.8] | 27.9 [23.1-32.7] | 22.5 [19.3-25.7] | 6.6 [3.1-10.1] | 27.5 [22.7-32.4] | 22.1 [18.9-25.3] | 4.5 [1.6-7.4] |
| All adults | ***n=540*** | ***n=1328*** | ***n=516*** | ***n=438*** | ***n=1216*** | ***n=490*** | ***n=463*** | ***n=1294*** | ***n=526*** | ***n=445*** | ***n=1259*** | ***n=520*** |
| Suicidal ideation | 17.1 [13.9-20.3] | 10.5 [8.9-12.1] | 2.9 [1.5-4.3] | 18.0 [14.4-21.6] | 11.0 [9.3-13.9] | 3.3 [1.7-4.9] | 16.7 [13.3-20.1] | 11.7 [10.0-13.4] | 3.3 [17.8-4.8] | 20.2 [16.5-23.9] | 11.0 [9.3-12.7] | 2.5 [14.3-20.9] |
| Suicide attempt | 1.1 [0.2-2.0] | 1.0 [0.5-1.5] | 0.0 [0-0] | 1.2 [0.2-2.2] | 0.8 [0.3-1.3] | 0.2 [-0.2-0.6] | 1.1 [0.5-2.1] | 1.2 [0.6-1.8] | 0.0 [0-0] | 1.6 [0.4-2.8] | 1.0 [0.5-1.6] | 0.0 [0-0] |
| Self- harm | 2.9 [1.5-4.3] | 1.5 [0.9-2.2] | 0.6 [-0.1-1.3] | 2.3 [0.9-3.7] | 1.2 [0.6-1.8] | 0.0 [0-0] | 4.0 [2.2-5.8] | 1.7 [1.0-2.4] | 0.2 [-0.2-0.6] | 2.3 [0.9-3.7] | 1.7 [1.0-2.4] | 0.2 [-0.2-0.6] |
| PHQ-9 (% ≥ 10) | 31.4 [27.5-35.3] | 22.8 [20.6-25.0] | 9.7 [7.2-12.3] | 29.7 [25.4-34.0] | 22.2 [19.9-24.5] | 9.2 [6.7-11.8] | 31.4 [27.2-35.6] | 23.4 [21.1-25.7] | 10.8 [8.2-13.5] | 37.2 [32.7-41.7] | 25.8 [23.4-28.2] | 11.0 [8.3-13.7] |
| GAD-7 (% ≥ 10) | 25.4 [21.7-29.1] | 17.8 [15.8-19.8] | 7.0 [4.8-9.2] | 22.9 [19.0-26.8] | 16.6 [14.5-18.7] | 6.1 [4.0-8.2] | 23.8 [19.9-27.7] | 17.9 [15.8-20.0] | 6.8 [4.7-9.0] | 25.9 [21.8-30.0] | 17.6 [15.5-19.7] | 6.7 [4.6-8.8] |

^a^ Last week, ^b^ Last 2 weeks

**Table S3: Recent suicidal history and depressive symptoms and anxiety symptoms cut-off scores by presence any mental health (MH) problems and socioeconomic grouping (SEG)**

| Variable | Wave 4: May/June 2020 (n=2384) | | Wave 5: July/August 2020 (n=2144) | | Wave 6: October 2020 (n=2283) | | Wave 7: February 2021 (n=2224) | |
| --- | --- | --- | --- | --- | --- | --- | --- | --- |
|  | % [95% CI] | % [95% CI] | % [95% CI] | % [95% CI] | % [95% CI] | % [95% CI] | % [95% CI] | % [95% CI] |
| Pre-existing mental health (MH) | **No MH (n=1762)** | **MH (n= 622)** | **No MH (n=1596)** | **MH (n=548)** | **No MH (n=1713)** | **MH (n=570)** | **No MH (n=1678)** | **MH (n=546)** |
| Suicidal ideation^a^ | 7.3 [6.1-8.5] | 19.3 [16.2-22.4] | 7.2 [5.9-8.5] | 21.0 [17.6-24.4] | 7.8 [6.5-9.1] | 20.0 [16.7-23.3] | 8.0 [6.7-9.3] | 19.6 [16.3-22.9] |
| Suicide attempt^a^ | 0.7 [0.3-1.1] | 1.2 [0.4-2.1] | 0.7 [0.3-1.1] | 0.8 [0.1-1.5] | 0.9 [0.5-1.3] | 0.9 [0.1-1.7] | 1.0 [0.5-1.5] | 0.6 [-0.1-1.3] |
| Self- harm^a^ | 0.7 [0.3-1.1] | 4.0 [2.5-5.5] | 0.6 [0.2-1.0] | 3.0 [1.6-4.4] | 1.5 [0.9-2.1] | 2.9 [1.5-4.3] | 1.1 [0.6-1.6] | 2.6 [1.3-3.9] |
| PHQ-9 (% ≥ 10)^b^ | 13.7 [12.1-15.3] | 45.2 [41.3-19.1] | 13.0 [11.4-14.6] | 43.6 [39.6-47.7] | 13.4 [11.8-15.0] | 48.2 [44.1-52.3] | 17.3 [15.5-19.1] | 47.4 [43.2-51.6] |
| GAD-7 (% ≥ 10)^b^ | 10.4 [9.0-11.8] | 36.2 [32.4-40.0] | 9.3 [7.9-10.7] | 33.6 [29.7-37.5] | 9.7 [8.3-11.1] | 37.2 [33.4-41.2] | 10.5 [9.1-12.0] | 35.7 [31.7-39.7] |
| Socio-economic grouping (SEG) | **High SEG (n=1362)** | **Low SEG (n=1022)** | **High SEG (n=1235)** | **Low SEG (n=909)** | **High SEG (n=1311)** | **Low SEG (n=972)** | **High SEG (n=1284)** | **Low SEG (n=940)** |
| Suicidal ideation | 9.8 [8.2-11.4] | 11.0 [9.1-12.9] | 9.6 [8.0-11.2] | 12.0 [9.9-14.1] | 10.5 [8.5-12.5] | 11.0 [9.0-13.0] | 10.1 [8.5-11.7] | 11.8 [12.4-16.9] |
| Suicide attempt | 0.7 [0.3-1.1] | 0.9 [0.3-1.5] | 0.8 [0.3-1.3] | 0.6 [0.1-1.1] | 1.2 [0.5-1.9] | 0.5 [0.1-0.9] | 1.2 [0.6-1.8] | 0.5 [0.1-1.0] |
| Self- harm | 1.3 [0.7-1.9] | 2.0 [1.2-2.9] | 1.1 [0.5-1.7] | 1.2 [0.5-1.9] | 1.9 [1.0-2.8] | 1.8 [1.0-2.6] | 1.7 [1.0-2.4] | 1.2 [0.5-1.8] |
| PHQ-9 (% ≥ 10) | 19.7 [17.6-21.8] | 25.0 [22.4-27.6] | 17.2 [15.1-19.3] | 25.7 [22.9-28.5] | 19.4 [16.8-22.0] | 25.7 [23.0-28.4] | 21.0 [18.8-23.2] | 29.8 [26.9-32.7] |
| GAD-7 (% ≥ 10) | 15.6 [13.7-17.5] | 19.3 [16.9-21.7] | 13.8 [11.9-15.7] | 17.7 [15.2-20.2] | 15.3 [13.0-17.6] | 18.3 [15.9-20.7] | 14.6 [12.7-16.5] | 19.5 [17.0-22.0] |

^a^ Last week, ^b^ Last 2 weeks

**Table S4. Defeat, entrapment, loneliness, and wellbeing mean scores and 95% confidence intervals [CIs] by age group and sex**

| Variable | Wave 4: May/June 2020 (n=2384) | | | Wave 5: July/August 2020 (n=2144) | | | Wave 6: October 2020 (n=2283) | | | Wave 7: February 2021 (n=2224) | | |
| --- | --- | --- | --- | --- | --- | --- | --- | --- | --- | --- | --- | --- |
|  | **18-29 years** | **30-59 years** | **60+ years** | **18-29 years** | **30-59 years** | **60+ years** | **18-29 years** | **30-59 years** | **60+ years** | **18-29 years** | **30-59 years** | **60+ years** |
|  | M [95% CI] | M [95% CI] | M [95% CI] | M [95% CI] | M [95% CI] | M [95% CI] | M [95% CI] | M [95% CI] | M [95% CI] | M [95% CI] | M [95% CI] | M [95% CI] |
| Men | ***n=141*** | ***n=651*** | ***n=325*** | ***n=117*** | ***n=601*** | ***n=308*** | ***n=129*** | ***n=635*** | ***n=329*** | ***n=120*** | ***n=621*** | ***n=322*** |
| Defeat | 3.97 [3.24-4.70] | 3.65 [3.34-3.96] | 1.89 [1.57-2.21] | 4.14 [3.35-4.93] | 3.44 [3.12-3.76] | 2.19 [1.84-2.54] | 4.13 [3.42-4.84] | 3.59 [3.28-3.90 | 2.42 [2.06-2.78] | 4.33 [3.54  -5.12] | 3.78 [3.47-4.09] | 2.45 [2.08-2.82] |
| Entrapment | 3.60 [2.89-4.31] | 3.43 [3.11-3.75] | 1.67 [1.34-2.00] | 3.89 [3.09-4.69] | 3.18 [2.84-3.52] | 1.76 [1.42-2.10] | 3.72 [2.98-4.46] | 3.34 [3.01-3.67] | 1.89 [1.53-2.25] | 4.30 [3.47-5.13] | 3.36 [3.03-3.69] | 2.04 [1.67-2.41] |
| Loneliness | 5.11 [4.81-5.41] | 4.96 [4.81-5.11] | 4.25 [4.07-4.43] | 5.09 [4.77-5.41] | 4.88 [4.73-5.03] | 4.24 [4.05-4.43] | 5.02 [4.70-5.34] | 4.86 [4.71-5.01] | 4.30 [4.11-4.49] | 5.33 [5.00-5.66] | 4.96 [4.81-5.11] | 4.37 [4.18-4.56] |
| Wellbeing | 22.60 [21.48-23.72] | 23.09 [22.59-23.59] | 26.70 [26.10-27.30] | 21.99 [20.74-23.24] | 23.36 [22.84-23.88] | 26.66 [26.05-27.27] | 22.86 [21.79-23.93] | 23.15 [22.65-23.65] | 26.02 [25.40-26.64] | 22.55 [21.34-23.76] | 23.45 [22.93-23.97] | 26.46 [25.8-27.10] |
| Women | ***n=398*** | ***n=676*** | ***n=191*** | ***n=320*** | ***n=614*** | ***n=182*** | ***n=333*** | ***n=658*** | ***n=197*** | ***n=324*** | ***n=637*** | ***n=198*** |
| Defeat | 5.13 [4.73-5.53] | 4.47 [4.15-4.79] | 2.80 [2.36-3.24] | 5.18 [4.70-5.66] | 4.54 [4.20-4.88] | 2.68 [2.25-3.11] | 5.48 [5.03-5.93] | 4.87 [4.54-5.20] | 2.90 [2.45-3.35] | 5.57 [5.10-6.03] | 5.06 [4.73-5.39] | 2.76 [2.34-3.18] |
| Entrapment | 4.58 [4.15-5.01] | 4.14 [3.80-4.48] | 2.30 [1.84-2.76] | 4.68 [4.18-5.18] | 4.24 [3.88-4.60] | 2.20 [1.7-2.64] | 5.26 [4.75-5.77] | 4.42 [4.07-4.77] | 2.54 [2.07-3.01] | 5.16 [4.65-5.67] | 4.56 [4.21-4.91] | 2.28 [1.85-2.71] |
| Loneliness | 5.71 [5.53-5.89] | 5.41 [5.26-5.56] | 4.73 [4.47-4.99] | 5.75 [5.55-5.95] | 5.33 [5.17-5.49 | 4.74 [4.47-5.01] | 5.75 [5.55-5.95] | 5.35 [5.20-5.50] | 4.62 [4.3-4.87] | 5.87 [5.67-6.07] | 5.50 [5.35-5.65] | 4.67 [4.42-4.92] |
| Wellbeing | 21.27 [20.64-21.90] | 22.65 [22.18-23.12] | 25.79 [25.02-26.56] | 21.22 [20.53-21.91] | 22.74 [22.24-23.24] | 25.72 [24.93-26.51] | 20.70 [20.01-21.39] | 22.35 [21.86-22.84] | 25.44 [24.66-26.22] | 20.97 [20.30-21.64] | 22.47 [21.99-22.95] | 26.00 [25.20-26.80] |
| All adults | ***n=540*** | ***n=1328*** | ***n=516*** | ***n=438*** | ***n=1216*** | ***n=490*** | ***n=463*** | ***n=1294*** | ***n=526*** | ***n=445*** | ***n=1259*** | ***n=520*** |
| Defeat | 4.83 [4.47-5.19] | 4.07 [3.85-4.29] | 2.23 [1.97-2.49] | 4.90 [4.49-5.31] | 4.00 [3.76-4.24] | 2.37 [2.10-2.64] | 5.10 [4.71-5.49 | 4.24 [4.01-4.47] | 2.60 [2.32-2.88] | 5.24 [4.84-5.64] | 4.43 [4.20-4.66] | 3.44 [2.29-2.85] |
| Entrapment | 4.32 [3.95-4.69 | 3.79 [3.55-4.03] | 1.90 [1.63-2.17] | 4.47 [4.05-4.89] | 3.71 [3.46-3.96] | 1.92 [1.65-2.19] | 4.83 [4.40-5.26 | 3.89 [3.65-4.13] | 2.13 [1.84-2.42] | 4.93 [4.49-5.37] | 3.97 [3.73-4.21] | 3.07 [1.85-2.41] |
| Loneliness | 5.56 [5.40-5.72] | 5.19 [5.09-5.29] | 4.43 [4.28-4.58] | 5.57 [5.40-5.74] | 5.11 [5.00-5.22] | 4.43 [4.27-4.59] | 5.54 [5.37-5.71] | 5.11 [5.00-5.22] | 4.42 [4.27-4.57] | 5.72 [5.55-5.89] | 5.23 [5.12-5.34] | 4.83 [4.33-4.63] |
| Wellbeing | 21.62 [21.07-22.17] | 22.87 [22.53-23.21] | 26.36 [25.89-26.83] | 21.43 [20.82-22.04] | 23.05 [22.69-23.41] | 26.31 [25.82-26.80] | 21.31 [20.72-21.90] | 22.75 [22.40-23.10] | 25.81 [25.32-26.30] | 21.40 [20.81-21.99] | 22.95 [22.60-23.30] | 24.26 [25.78-26.78] |

**Table S5. Defeat, entrapment, loneliness, and wellbeing mean scores and 95% confidence intervals [CIs] by age group and sex**

| Variable | Wave 4: May/June 2020 (n=2384) | | Wave 5: July/August 2020 (n=2144) | | Wave 6: October 2020 (n=2283) | | Wave 7: February 2021 (n=2224) | |
| --- | --- | --- | --- | --- | --- | --- | --- | --- |
|  | M [95% CI] | M [95% CI] | M [95% CI] | M [95% CI] | M [95% CI] | M [95% CI] | M [95% CI] | M [95% CI] |
| Pre-existing mental health (MH) | **No MH (n=1762)** | **MH (n= 622)** | **No MH (n=1596)** | **MH (n=548)** | **No MH (n=1713)** | **MH (n=570)** | **No MH (n=1678)** | **MH (n=546)** |
| Defeat | 2.90 [2.74-3.06] | 6.52 [6.17-6.87] | 2.88 [2.71-3.05] | 6.54 [6.16-6.92] | 3.08 [2.91-3.25] | 6.94 [6.58-7.30] | 3.26 [3.09-3.43] | 6.94 [6.57-7.31] |
| Entrapment | 2.51 [2.35-2.67] | 6.32 [5.93-6.71] | 2.50 [2.33-2.67] | 6.28 [5.87-6.69] | 2.66 [2.49-2.83] | 6.75 [6.34-7.16] | 2.82 [2.64-3.00] | 6.56 [6.15-6.97] |
| Loneliness | 4.80 [4.72-4.88] | 5.99 [5.83-6.15] | 4.72 [4.63-4.81] | 6.01 [5.84-6.18] | 4.71 [4.62-4.80] | 6.01 [5.85-6.17] | 4.85 [4.76-4.94] | 6.10 [5.94-6.26] |
| Wellbeing | 24.60 [24.32-24.88] | 19.73 [19.24-20.22] | 24.90 [24.45-25.05] | 19.69 [19.17-20.21] | 24.43 [24.14-24.72] | 19.31 [18.81-19.81] | 24.51 [24.21-24.81] | 20.05 [19.53-20.57] |
| Socio-economic grouping (SEG) | **High SEG (n=1362)** | **Low SEG (n=1022)** | **High SEG (n=1235)** | **Low SEG (n=909)** | **High SEG (n=1311)** | **Low SEG (n=972)** | **High SEG (n=1284)** | **Low SEG (n=940)** |
| Defeat | 3.49 [3.29-3.69] | 4.32 [4.05-4.59] | 3.37 [3.16-3.58] | 4.42 [4.13-4.71] | 3.64 [3.43-3.85] | 4.59 [4.31-4.87] | 3.75 [3.54-3.96] | 4.72 [4.44-5.00] |
| Entrapment | 3.19 [2.98-3.40] | 3.93 [3.65-4.21] | 3.13 [2.91-3.35] | 3.92 [3.62-4.22] | 3.32 [3.10-3.54] | 4.17 [3.88-4.46] | 3.40 [3.18-3.62] | 4.19 [3.89-4.49] |
| Loneliness | 4.99 [4.89-5.09] | 5.27 [5.15-5.39] | 4.90 [4.80-5.00] | 5.24 [5.11-5.37] | 4.90 [4.80-5.00] | 5.22 [5.09-5.35] | 5.01 [4.91-5.11] | 5.35 [5.22-5.48] |
| Wellbeing | 23.68 [23.35-24.01] | 22.87 [22.46-23.28] | 23.94 [23.59-24.29] | 22.80 [22.36-23.24] | 23.60 [23.26-23.94] | 22.55 [22.13-22.97] | 23.85 [23.50-24.20] | 22.83 [22.41-23.25] |

Figure S1: Changes in wellbeing scores (mean) over waves 1 – 7 of the UK COVID-19 Mental Health and Wellbeing study


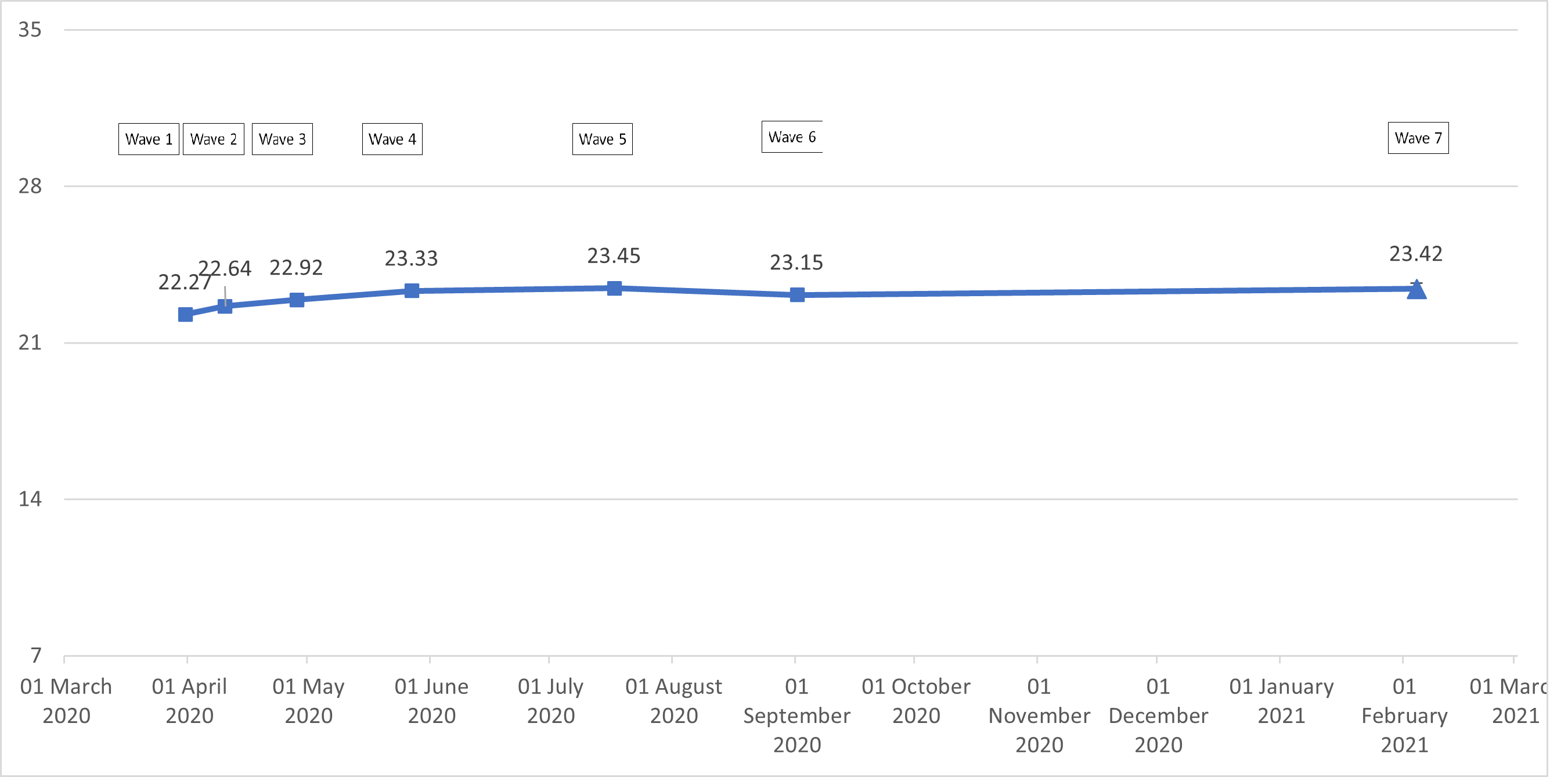

Supplement: Supplementary file 1 [file S2056472422000588sup001.docx]
